# A Review of Ultrafine-Grained Magnetic Materials Prepared by Using High-Pressure Torsion Method

**DOI:** 10.3390/ma15062129

**Published:** 2022-03-14

**Authors:** Zhi-Rui Wang, Ping-Zhan Si, Jihoon Park, Chul-Jin Choi, Hong-Liang Ge

**Affiliations:** 1 College of Materials Science and Chemistry, China Jiliang University, Hangzhou 310018, China; wangzhirui9264@163.com; 2 Powder and Ceramic Division, Korea Institute of Materials Science, Changwon 51508, Korea; jpark@kims.re.kr (J.P.); cjchoi@kims.re.kr (C.-J.C.)

**Keywords:** high-pressure torsion, magnetic materials, ultrafine grain, severe plastic deformation, nanocrystalline, coercivity, strain, structure

## Abstract

High-pressure torsion (HPT) is a severe plastic deformation technique where a sample is subjected to torsional shear straining under a high hydrostatic pressure. The HPT method is usually employed to create ultrafine-grained nano-structures, making it widely used in processing many kinds of materials such as metals, glasses, biological materials, and organic compounds. Most of the published HPT results have been focused on the microstructural development of non-magnetic materials and their influence on the mechanical properties. The HPT processing of magnetic materials and its influence on the structural and magnetic properties have attracted increasing research interest recently. This review describes the application of HPT to magnetic materials and our recent experimental results on Mn_3_O_4_, Mn_4_N, and MnAl-based alloys. After HPT, most magnetic materials exhibit significantly reduced grain size and substantially enhanced coercivity.

## 1. Introduction

The high-pressure torsion (HPT) method, first introduced by Percy W. Bridgman in 1935 [1], has received extensive attention as a severe plastic deformation (SPD) technique in creating ultrafine-grained structures with novel physical properties [2]. The term ‘ultrafine-grained’ is usually defined as having fully homogeneous and equiaxed microstructures with average grain sizes of less than ~1 μm and with a majority of the grain boundaries having high-angles of misorientation [3]. A number of metals and alloys have been processed by HPT to investigate new high-performance materials. HPT produces high-density crystal defects and grain boundaries, which, acting as pinning points in preventing dislocation motion, leading to the improved strength of materials. The grain size of a material usually determines the physical properties of the material. Strain hardening (i.e., strengthening of a material by plastic deformation) and anomalous magnetic properties were frequently observed in a number of materials such as Cu and Fe processed by HPT, respectively [4,5]. Although a large number of reports are now available describing the application of HPT processing to a wide range of materials for grain refinement, most of the published results have been focused on the microstructural development of non-magnetic materials and their influence on the mechanical properties as expected from Hall–Petch equation. In comparison, the studies on the application of HPT to the magnetic materials are limited, even though the magnetic properties, coercivity (*H_c_*), for instance, of ferromagnetic materials are highly dependent on the grain size of the materials. The *H_c_* of a magnetic material is known to be proportional to D^−1^ when the grain size D exceeds the ferromagnetic exchange length, while *H_c_* becomes proportional to D^6^ when D is smaller than the critical grain size of the ferromagnetic exchange length [6,7].

Magnetic nanocrystalline alloys in the form of ribbons are usually prepared by using rapid quenching method and/or annealing of the amorphous alloys [8,9]. Magnetic nanoparticles prepared by chemical and/or physical methods have been extensively studied for more than two decades [10]. There are far fewer reports on magnetic nanocrystalline alloys, oxides, and nitrides prepared by HPT. However, the HPT processing of magnetic materials has attracted increasing research interests for the high efficiency of HPT in producing ultra-fine grain size, which is crucial for tuning the magnetic properties of the magnetic materials [11,12,13]. This review presents a summary of the background and the recent developments of the magnetic materials processed by HPT.

Besides the summary of the previously published work on magnetic materials prepared by HPT, our recent research results are also presented in this review. The magnetic properties of Mn_3_O_4_ and Mn_4_N processed by HPT at room temperature have been studied systematically. After HPT, the grain size of both Mn_3_O_4_ and Mn_4_N was reduced significantly while the coercivity of them was enhanced significantly. HPT can not only be employed to process metallic materials but also can be used for processing of oxides and nitrides.

## 2. Principles of High-Pressure Torsion

### 2.1. Historical Background of HPT

The early scientific report on high-pressure torsion can be traced back to 1935, when Bridgman expounded the fundamental concept of high-pressure torsion and carried out a series of experimental studies on bulk materials processed under high pressure [1]. Bridgman described his idea “If a bar is twisted while a longitudinal compressive load is simultaneously applied, it is possible to twist the bar through much greater angles without fracture than is possible without the compressive load. At the same time the magnitude of the torque which the bar can support without fracture is increased” [14]. The Nobel Prize in Physics 1946 was awarded to Percy Williams Bridgman “for the invention of an apparatus to produce extremely high pressures, and for the discoveries he made therewith in the field of high-pressure physics” [3]. HPT have been mainly employed to enhance the mechanical properties, such as hardness and yield strength of materials. In 1988s, Valiev reported the unusually high elongation in an Al-4% Cu-0.5% Zr alloy after processing by HPT to a strain at 7 [15]. After that, a series of development and research works on HPT technology were conducted in Russia. HPT has been used as a machining tool with remarkable grain refinement ability and enhanced mechanical properties of metals. In 1991, Valiev et al. obtained submicron grain size nanostructures by using pure shear in experiments on pure copper. They studied the phase transformation and the change of organization structure of copper and found that after severe torsional deformation under high pressure, a uniform nanostructure with large-angle grain boundaries was formed inside the material, and the properties of the material also changed qualitatively [16]. High-pressure torsion may also serve as an alternative method for producing bulk ultrafine grained materials. Magnetic materials processed by HPT usually exhibit ultrafine-grained structure with enhanced coercivity, decreased saturation magnetization, and improved magnetic energy product. Most solid materials, including pure metals, metallic alloys, composite materials, oxides, and nitrides, etc., could be processed by HPT.

### 2.2. Devices and Parameters of High-Pressure Torsion

The HPT equipment developed at early time was relatively simple; the torsion function relied on manual wrenching back and forth to achieve the center plate [2]. Several scientists had improved the high-pressure torsion equipment later in their work. In 1960, Griggs built a device similar to the current HPT, which used one pair of anvils [17]. The lower anvil was fixed, and the upper anvil was rotated by a motor to produce shear strain [18]. In the early 1990s, the machine installed by Valiev used a continuous rotation of the anvil driven by an electric motor [19]. 

**Figure 1:**
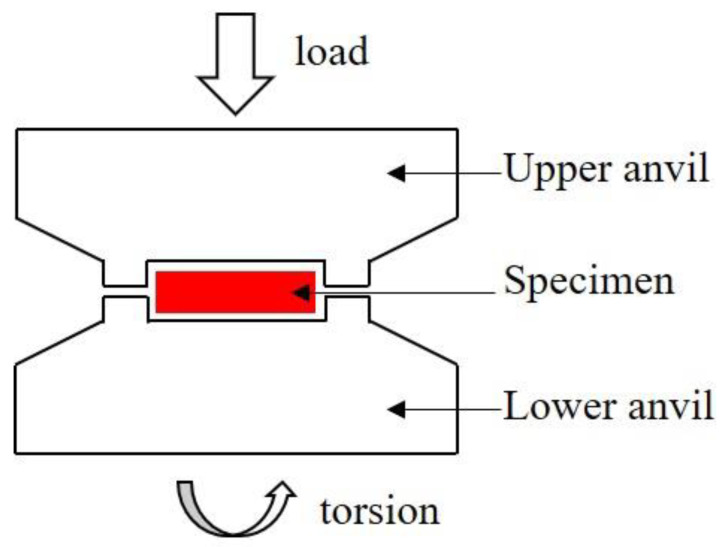
Schematic illustration of the HPT devices.

The schematic diagram of most HPT devices is shown in Figure 1. A disk specimen is first loaded into the space between two anvils and then pressed under high pressure. A torsional strain is imposed through the rotation of the lower or the upper anvil. Torque is generated on the cross section of the sample through the friction between the contact surfaces of the anvils and the sample. Tangential shear deformation occurs inside the sample until the set number of turns is completed. The high-pressure torsion generates a huge pressure environment, friction force, and shear force in the deformation zone of the sample. These forces are used to refine the crystal grains. It should be noted that the pressure distribution in the HPT disk sample was uneven due to size effects, friction effects, and material flow [2].

The load force applied to the sample is generally denoted by kN, which can be divided by the contact area between the sample and the anvil head to obtain a pressure in GPa. One anvil rotates in a pre-set number of revolutions and torsion speeds. The applied torque induces strains in the sample. The strain effect in HPT is most influenced by the number of torsional turns N and the radius of the specimen disc. For the same sample, the strain increases gradually from inside to outside in the radial direction. For the same position, the strain increases with more turns of torsion. The torsional deformation of the sample disc is achieved by the surface friction between the sample and the anvils. Therefore, the magnitude of the frictional force is also a factor to be considered during HPT. If there is poor contact between the surfaces of the material and the anvils, there is a high risk of slippage during the experiment, resulting in poor strain. It is worth noting that the angular velocity of rotation is also a factor that can affect the magnitude of strain. To ensure a good contact between the thin sample and the anvils, a low angular velocity is generally used.

## 3. Application of HPT to Pure Magnetic Elements

Iron, cobalt, and nickel are well-known ferromagnetic transition metals. The application of HPT to Fe, Co, and Ni was first reported by Bridgman in 1935. These pure metals rotated perfectly smoothly during HPT. The shearing strength of Fe, Co, and Ni at 42,000 kg/cm^2^, 50,000 kg/cm^2^, and 50,000 kg/cm^2^ was reported to be 10,600 kg/cm^2^, 6300 kg/cm^2^, and 8700 kg/cm^2^, respectively [1]. The Vickers hardness of Fe after HPT at room temperature was measured to be ~530 [20]. Although the mechanical properties of Fe, Co, and Ni have been studied since the invention of HPT device, the study of the magnetic properties of these ferromagnetic metals after HPT can only be found after 1990s.

Different from that of pure Fe without HPT, two states of Fe atoms with different parameters of the electric and magnetic hyperfine structure were found, in the Mössbauer experiments, to exist in the iron prepared by HPT. One state coincides with that in the conventional iron. Another state corresponds to the state of the intercrystalline grain boundary phase [19]. Y. Oba and co-workers reported nanoscale spin dislocations formed in the pure iron treated by HPT. The spin misalignment is not consistent with grain size and may only form around the high-density defects and grain boundaries created by HPT. The spin misalignment can be observed in magnetic fields up to 10 T, indicating that anomalous magnetic anisotropy is induced in the HPT-Fe [5]. The saturation magnetization of Fe varies little after HPT [7]. However, the coercivity of Fe was enhanced significantly by more than 12 times after HPT (Table 1), owing to the decrease of the grain size [20]. In Table 1, we have also listed the coercivities of a number of other magnetic materials before and after HPT.

HPT not only results in changes of the grain size, magnetic properties, mechanical properties, and domain structure, but also induces phase transformation in cobalt. The grain size of Co decreases from 2 mm to 120 ± 65 nm, while the hardness of Co is doubled after HPT [28]. The magnetic coercivity and magnetic retentivity of Co increase substantially when the grain size is decreased by HPT. When the average grain size reaches the submicron level, Co undergoes a phase transformation from metastable fcc-Co to hcp-Co. Nevertheless, when the grain size of Co is reduced to the nanoscale, the phase transition hcp→fcc occurs [28]. TEM micrographs show that the {111} deformation twins were formed within the fcc nanograins. Room temperature HPT processing to cast Co (99.3% Co) under 6 GPa resulted in sub-micron scale Co (~0.1 µm) and a different domain structure from that of the coarse-grained (10 µm) specimens [27]. The decrease of grain size slightly changes the form of the domain structure of a striped type, with each domain spreading all over a great number of microcrystallites. Such a domain structure is similar to the domain structure of monocrystals with an easy magnetization tilt axis, which evidently is due to the texture formed during the specimen deformation [29]. Unlike a strongly deformed one, the coarse-grained Co forms the regular-striped domain structure in each separate grain (crystallite), and, near some grain boundaries, domain walls join with domain walls of adjacent grains [29].

The HPT treatment of commercially pure nickel (99.6%) at liquid nitrogen temperature resulted in a nanostructure with an average grain size of 80 nm and a microhardness of up to 6200 MPa [30]. HPT strain was found to lead to spin misalignment in pure Ni by magnetic field-dependent unpolarized small-angle neutron scattering experiments and remains present in magnetic fields up to 4 T. The spin-misalignment scattering patterns are elongated perpendicular to the applied magnetic field due to the predominance of unusual longitudinal sin^2^θ-type angular anisotropy [31]. Different from Fe and Co, HPT has a more complicated effect on the magnetic properties of Ni. The coercivity of the HPT-ed Ni depends on the length of time that the sample is kept at room temperature. The coercivity of Ni in a highly strained state can be significantly increased up to 5.12 kA/m [27]. In another report, the Hc of Ni increased slightly after HPT. The high magnetostriction in Ni dominates the magnetic behaviors of Ni [20].

Among the rare earth metals, gadolinium is the only metal that is ferromagnetic at room temperature. A submicrocrystalline two-phase structure was formed in the coarse-grained Gd after HPT. A lower magnetization value was observed over the whole temperature range of Gd ferromagnetism after HPT. The substantial increase in coercivity can be attributed to the fine and dispersed internal formation of heterogeneous structures. The temperature dependence of the paramagnetic susceptibility demonstrates that the effective atomic magnetic moment was significantly reduced after HPT [32].

Interestingly, in pure hafnium (Hf), a typical paramagnet, severe plastic deformation by HPT may trigger room temperature ferromagnetism [33]. The spontaneous magnetization in HPT-ed pure Hf at room temperature is due to the generation of a new monoclinic crystal structure, which is caused by the induction of elastic lattice distortion due to the refinement of the grains to the nanoscale by HPT. The severe plastic deformation may be explored to tune the magnetic properties and, in particular, to induce room temperature ferromagnetism in bulk non-magnetic metals [33].

## 4. Application of HPT to Binary Magnetic Alloys

### 4.1. Fe-Based Binary Alloys

Several Fe-based magnetic binary alloys, including Fe-C, Fe-Si, Fe-Al, Fe-Cu, Fe-Ni, and Fe-Co, have been processed by HPT. The Fe-C alloys (C from 0.05 to 1.7 wt%) in the as-cast state and after HPT at ambient temperature and 5 GPa with five anvil rotations were studied [34]. HPT leads to the grain refinement, the disappearance of the non-equilibrium phases, and the formation of the phases, which are equilibrium at temperature and pressure of HPT treatment. After HPT, only Fe_3_C and α-Fe were detected in the alloy. It seems the saturation magnetization of Fe-C alloys does not vary too much after HPT. However, the saturation magnetization of HPT alloys decreases with increasing carbon content slower than that of as-cast alloys [34]. Fe-C alloys after HPT have nanostructures with higher coercivity than that of the coarse-grained cast alloys [21].

Besides grain size refinement, HPT can also be employed to produce immiscible alloys. The immiscible Fe-Cu system was prepared by HPT from high purity Fe and Cu powders [22,35]. In the as-deformed states, Fe exists in the form of clusters and diluted state in the fcc-Cu matrix. As the composition of the alloy changes, the ratio of clustered and diluted Fe in the alloy changes. Such a change causes either thermal relaxation (in case of Fe25wt%-Cu75wt%), magnetic frustration (in case of Fe7wt%-Cu93wt%), or a superposition of both (in the case of Fe14wt%-Cu). The enhanced coercivity as a function of the annealing temperature complies with the random anisotropy model, showing that Fe-particle sizes below the exchange length persist in annealed states. The experimental results show that HPT is effective in mixing the two components at the atomic scale [22]. An ingot ring of Cu78wt%-22wt%Fe alloy was subjected to HPT for varied rotating revolutions. After 10 turns of HPT treatment, the Vickers microhardness increased from ~160 Hv in the as cast state to ~400 Hv. The saturation magnetization of Cu78wt%-22wt%Fe decreases while the coercivity increases with increase in HPT rotations [23].

HPT can also be employed to produce phases that are stable at lower temperatures but are unstable at higher temperatures. The L1_0_-ordered FeNi phase is found to be stable at temperatures down to 320 °C. Therefore, it is very difficult to produce the L1_0_-ordered phase manually by conventional annealing on a practical time scale. This is because the diffusion between atoms is very slow at low temperatures. However, it is possible to prepare L1_0_-ordered FeNi phase by mixing Fe and Ni powders and then using HPT treatment. A high-density of lattice defects is introduced inside the sample by severe deformation, which leads to enhanced atomic diffusion resulting in the formation of the L1_0_ phase [36]. However, the situation is completely different when a small amount of Ti is added to FeNi, and no L1_0_ structure is generated inside the sample even after HPT treatment [37].

HPT may also induce variation of the magnetic states of Fe-Al alloys. A paramagnetic to ferromagnetic transition upon plastic deformation by HPT was observed in the B2 ordered Fe-28.3wt% Al specimens [38]. The reason for this change is the increase in the number of Fe-Fe nearest neighbor pairs, which is caused by the formation of high-density antiphase boundary tubes. The ferromagnetic state is a metastable one and vanishes upon heating at a temperature that is well below the temperature of re-ordering or dislocation recovery. HPT and certain deformation parameters result in the complete suppression of long-range order in the DO_3_-type Fe-13.2wt% Al alloy and in the corresponding increase in the Ms by 11% with respect to the equilibrium state [39].

The results of neutron diffraction and EXAFS spectroscopy tests show that HPT causes a significant reduction in the degree of long-range order in FeCo alloys. It is demonstrated that, after HPT, an ultrafine structure with a high degree of B2 ordering was formed in the FeCo alloy covering the nearest coordination spheres of Fe and Co atoms with a very low intensity of superstructure lines in neutron diffraction patterns [40].

The coercivity of Fe-3wt%Si increased by a factor of about 14 after room temperature HPT, and that of Fe-6.5wt%Si increased by a factor of about 8 after room temperature HPT. The significant increase in coercivity after HPT also reflects the increase in internal stress after treatment, which is reflected in the increase in Vickers hardness. The HV decreases with increasing HPT temperature. Fe-6.5wt%Si exhibits the highest Vickers hardness values [20].

### 4.2. Co-Based Binary Alloys

HPT is not only used for tuning the structure and properties of Co-Cu, but also used for preparation of Co-Cu samples with Co and Cu powders [41]. Cu-rich alloys containing 10wt% ferromagnetic Co were treated using high-pressure torsion. The grains were significantly refined by HPT. The Ms decreases with straining and Hc increases with straining, but they level off after severe straining [23]. After processing of Cu-10wt%Co with HPT, magnetoresistance with an isotropic feature corresponding to giant magnetoresistance (GMR) appears at room temperature. The HPT is useful not only for controlling magnetic properties such as Ms and Hc but also for creating GMR in the alloys prepared by conventional ingot metallurgy [23,42].

The Cu–Co alloy with 2.2 and 4.9 wt% Co shows a much lower coercivity, even after HPT, as shown in Table 1 [25]. In another work on Co-rich alloys, the cast Co-5.6wt% Cu and Co-13.6 wt% Cu alloys were subjected to severe plastic deformation by HPT, which reduced the grain size of Co and the Cu precipitates significantly. As a result, the Hc of both the alloys radically increased significantly, as shown in Table 1. However, after HPT, the Ms remained nearly unchanged [26]. The magnetic properties of the Cu_74_Co_26_ alloy can be further tuned by additional HPT at liquid nitrogen temperature [43].

### 4.3. Ni-Based Binary Alloys

The nanocrystalline state induced by HPT in Ni_3_Al has been studied in detail [44,45]. The average grain size of Ni_3_Al and Ni_3_Al doped with Fe (7.5 at%) and Co (8 at%) after HPT using 10 revolutions and 10 GPa was estimated to be 20~30 nm. After HPT, a significant decrease took place in both the magnetic susceptibility of the Ni_3_Al alloy and magnetization of the Co-doped Ni_3_Al intermetallic compound. At room temperature, the initially ferromagnetic Fe-doped Ni_3_Al alloy became paramagnetic [46]. It is interesting that the coercivity for FeNi_3_ after HPT did not change with the significant grain size refinement, even though the Vickers microhardness of FeNi_3_ increased significantly after HPT [7].

### 4.4. Mn-Based Binary Alloys

**Figure 2:**
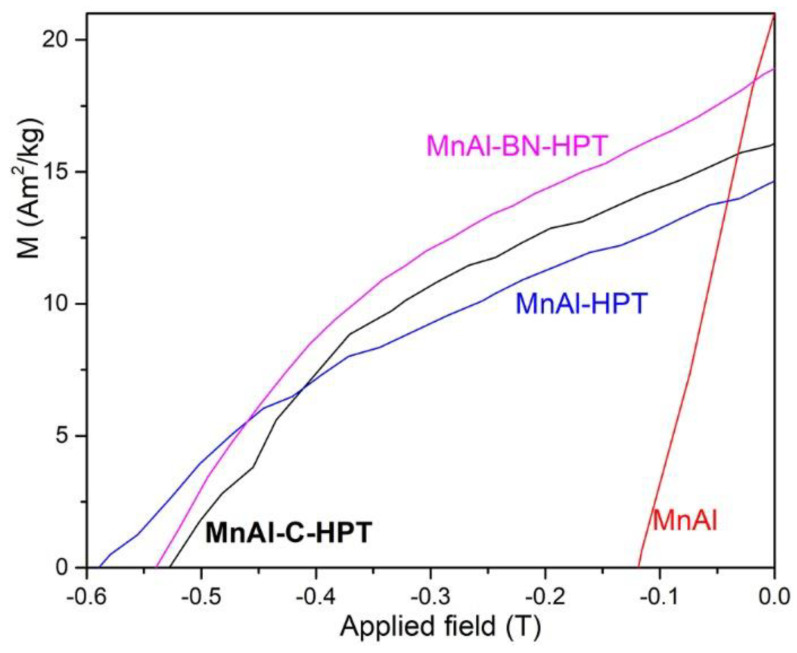
The demagnetization curves of the undeformed MnAl and the HPT-deformed MnAl, MnAl-C, and MnAl-BN, respectively.

Only recently, HPT was employed to process Mn-based rare-earth free magnetic materials. A record-high coercivity of 0.59 T in bulk MnAl disc was obtained by HPT [24]. The low coercivity achieved in MnAl alloys has become a barrier to developing high-performance MnAl-based magnets. The grain size of MnAl is significantly reduced while the *H_c_* is enhanced to 5 times that of the original sample when the ferromagnetic MnAl were severely deformed by HPT. The thermal-aging process enhances the magnetization of the deformed τ-phase MnAl significantly and reduces the coercivity of the MnAl disc slightly. The high coercivity of the sample is caused by a large number of pinning sites generated by HPT [24]. It is well-known that C could stabilize the meta-stable ferromagnetic MnAl phase. Here, we prepared ferromagnetic Mn_54_Al_46_C_2_._44_ (hereafter MnAl-C) for HPT experiments, after which the coercivity reached up to 0.52 T, which is slightly lower than the 0.59 T obtained in MnAl, owing to the presence of a fraction of non-magnetic phase in the MnAl matrix, acting as pinning center for magnetization reversal, as shown in Figure 2. A similar HPT process on Mn_53_Al_45_C_2_ produced an *H_c_* of 0.37 T [47]. Usually, the *H_c_* of the carbon-doped MnAl after HPT is lower than that of the carbon-free MnAl after HPT. We speculate that more pinning sites for magnetization reversal may be produced in meta-stable MnAl for the presence of a larger fraction of Mn-rich and/or Al-rich phase(s) in MnAl than that in MnAl-C. Carbon can stabilize the meta-stable ferromagnetic phase, and thus the formation of Mn-rich and/or Al-rich phase(s) in MnAl-C is inhibited. The BN-dopped MnAl (hereafter MnAl-BN) showed an *H_c_* of 0.54 T and a higher remanent magnetization after HPT [48]. The high coercivity developed in MnAl-based materials by HPT is potentially important in producing MnAl-based magnet. Deformation of MnAl by HPT leads to an increase in microhardness from 450 HV to 600 HV, which is obviously caused by the accumulation of deformation defects [49].

## 5. Application of HPT to Ternary Magnetic Alloys and Intermetallic Compounds

Several magnetic intermetallic compounds with a composition composed of three or more elements, especially Nd-Fe-B, have been processed by HPT and studied in detail. The application of HPT to Nd-Fe-B can not only improve the magnetic performance but also consolidate bulk magnets directly from powder compacts without sintering process. When compared to Nd_9_Fe_85_B_6_ magnets that were directly annealed without HPT, the remanent magnetization of α-Fe/Nd_2_Fe_14_B prepared by HPT and subsequent heat-treatment is improved by 13%, the coercivity by 19%, and the (*BH*)_max_ by 30%. The smaller grain size of α-Fe and Nd_2_Fe_14_B nanocrystals is the main reason for the enhanced magnetic properties [50]. After HPT, the coercivity of the magnet is enhanced due to the increase in domain-wall-pinning strength [51]. More interestingly, the amorphous state of Nd_9_Fe_85_B_6_ exhibits a completely different crystallization behavior after room temperature HPT, and the α-Fe nanocrystals, which smaller than 10 nm, were observed in amorphous matrix. Besides, it was found that HPT can effectively inhibit the formation of nonequilibrium magnetically soft phases (Nd_2_Fe_23_B_3_, Fe_3_B, and NdFe_7_) and increase the volume fraction of α-Fe in the magnet [51,52]. In addition, HPT induces the formation of α-Fe and Nd_2_Fe_14_B nanocrystals in amorphous matrix and suppresses the intermediate phase formation in the subsequent thermal annealing process. The volume fraction of α-Fe phase increases with increasing strain, but the grain size of the α-Fe phase and the Nd_2_Fe_14_B phase decreases significantly. As a result, the bulk magnets made at ε~6.2 exhibited (*BH*)_max_ = 17.8 MGOe and *H_c_* = 7.2 kOe, which is higher than the (*BH*)_max_ = 12.2 MGOe and *H_c_* = 6.2 kOe of the directly annealed (Nd-Pr)-Fe-Co-Nb-B [53]. Usually, the nanocrystallization of amorphous alloys is achieved by thermal annealing at elevated temperatures. This study shows another pathway to control the nanocrystallization of amorphous alloys. A relative density up to 90% of an Nd-Fe-B/α-Fe nanocomposite magnet having the coercivity 4.4 kOe and the remanence/saturation magnetization 100/165 emu/g was achieved after HPT of powders [54]. After HPT of an as-cast Fe-12.3at%Nd-7.6at%B alloy, the micro-structure contained the nanograins of the ferromagnetic Nd_2_Fe_14_B phase embedded in an amorphous matrix with uniform composition. In contrast, the commercial, multi-component, FeNdB-based alloy formed two different amorphous phases after HPT [55,56]. The HPT deformation at room temperature is usually accompanied by the formation of the shear bands, which leads to a texture that is usually uncontrollable in the magnetically hard phase [57]. Near the shear zone, both the hard magnetic Nd_2_Fe_14_B and soft magnetic α-Fe phases are distorted significantly, even though the crystallographic orientations of the Nd_2_Fe_14_B grains are randomized therein [53]. The coercivities of selected ternary magnetic alloys are listed in Table 2.

Co_80_Zr_20_ is a permanent magnet material that does not contain rare earths to which boron is added to increase the coercivity of the compound. After HPT for Co_80_Zr_16_B_4_, the coercivity increased to 2.25 kOe, owing to the grain refinement during HPT [59]. HPT of the initially annealed partially crystalline Hf_2_Co_11_B alloy led to its amorphization. The annealed Hf_2_Co_11_B subjected to HPT was characterized by the reduced coercive field, from 0.7 to 0.2 kOe, then the coercivity increase to 1.3 kOe due to the subsequent reannealing of the deformed sample. The method combining HPT and heat treatment was found to allow tuning of the structure and improving the hard magnetic properties of Hf_2_Co_11_B. The Ms of Hf_2_Co_11_B after HPT treatment is 679 emu/cm^−3^, which is 7% higher than that of the quenched condition [60]. HPT could not only refine the grain size of crystals but also could induce partial crystallization of some amorphous materials. When performing HPT for Fe_73.9_Cu_1_Nb_3_Si_15.5_B_6.6_, the formation of crystals crystallite with sizes ~3 nm at maximum was induced in the as-quenched amorphous alloy. It leads to a significant increase in the coercivity and magnetic anisotropy along with a decrease in the anisotropy energy density. In addition, HPT might be used to fabricate crack-free bulk samples from Fe_73.9_Cu_1_Nb_3_Si_15.5_B_6.6_ melt-spun ribbons [61]. The initially amorphous Fe-Ni-B alloys remain amorphous after HPT at 77 K, while HPT at 293 K leads to a partial crystallization of the amorphous state [62,63]. The saturation magnetization and coercivity of Fe-Ni-B change significantly after HPT processing with respect to the initial state. The sharp increase in the coercivity after HPT at 77 K is explained by the absence of relaxation channels for elastic stresses due to the suppression of thermal activation processes [63]. HPT causes nano-crystallization in amorphous Fe_78_Si_13_B_9_ with formation of Fe(Si) nanocrystals, leading to an increase in Ms by 40% [64]. The number of revolutions by HPT has significant effect on the magnetic properties and microhardness of Ni_44_Fe_29_Co_15_Si_2_B_10_, which shows a maximum increment of the saturation magnetization of 300% after HPT for 1 revolution. For Fe_50_Ni_33_B_17_ and Fe_70_Cr_15_B_15_, the M_s_ increased 120% and decreased 250%, respectively [65]. The Curie temperature, saturation magnetization, and coercivity field of the Ni_57_Fe_18_Ga_25_ shape memory alloy decrease when the applied deformation level by HPT is increased [66].

The HPT to SUS316L austenitic stainless steel resulted in phase transformation from austenitic γ→α’-martensite. Both M_s_ and *Hc* of the SUS316L were enhanced significantly after HPT, owing to the phase transformation and the grain refinement [67]. At a large Co/Fe ratio, Cu–Fe–Co formed nanocomposites after HPT. The soft magnetic properties of the alloy were verified, and the microstructure of the alloy remained stable at 400 °C [68]. The HPT treatment to Fe_50_Pd_46_Ni_4_ and Fe_50_Pd_42_Ni_8_ alloys resulted in an increase in Hc up to 1.52–2 times higher than the coercivity of the samples without HPT treatment and more than 5 times higher than the coercivity of the cast alloys [69].

## 6. Application of HPT to Magnetic Oxides

We have recently prepared nanocrystalline Mn_3_O_4_ bulk discs by using HPT under a load of 300 kN from commercial Mn_3_O_4_ powders at room temperature. The schematic diagram of the HPT device is shown in Figure 1. The grain size of Mn_3_O_4_ was decreased from over several tens of micrometers to ~30 nm, while the coercivity of Mn_3_O_4_ was increased from ~0.538 T to ~1.007 T, indicating high efficiency of the high-pressure torsion method in modifying the grain size and thus the magnetic properties of the samples. An exchange bias up to 148 mT has been observed in the severely deformed Mn_3_O_4_ samples.

**Figure 3:**
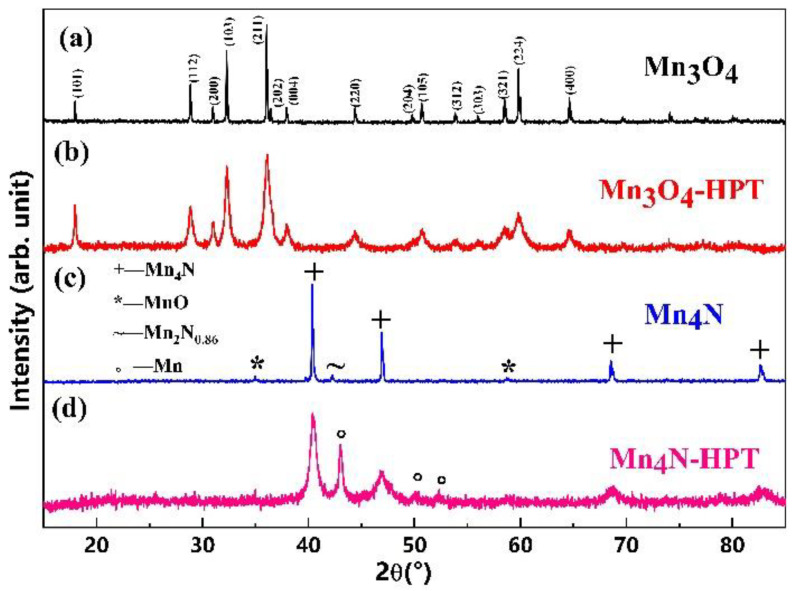
The XRD patterns of the Mn_3_O_4_ precursor (**a**), and the Mn_3_O_4_ after HPT with the numbers of rotations *n* = 3 (**b**), Mn_4_N precursor (**c**), and the Mn_4_N after HPT with the numbers of rotations *n* = 3 (**d**).

The structure of the precursor and the severely deformed Mn_3_O_4_ was characterized by using a Smartlab SE XRD diffractometer with Cu Kα radiation and a scanning angular step of 0.02°. Figure 3a,b shows the XRD patterns of the Mn_3_O_4_ precursor and the samples after HPT, respectively. The XRD patterns of the precursor could be indexed with Mn_3_O_4_ very well, as shown in Figure 3a, in which most diffraction peaks of Mn_3_O_4_ are narrow and sharp, indicating large crystalline size of Mn_3_O_4_ phase in the precursor, and this has been further proved by the subsequent SEM observations. No diffraction peaks from other impurities could be found in Figure 3a, indicating high purity of the precursor. The XRD patterns of the Mn_3_O_4_ samples after HPT could be indexed with Mn_3_O_4_ as well, as shown in Figure 3b. However, the diffraction peaks for the severely-deformed Mn_3_O_4_ are broadened significantly, indicating crucial grain size refinement during the deformation process. According to the Scherrer equation, the grain size of the HPT Mn_3_O_4_ samples is estimated to be in the range of 15~50 nm.

**Figure 4:**
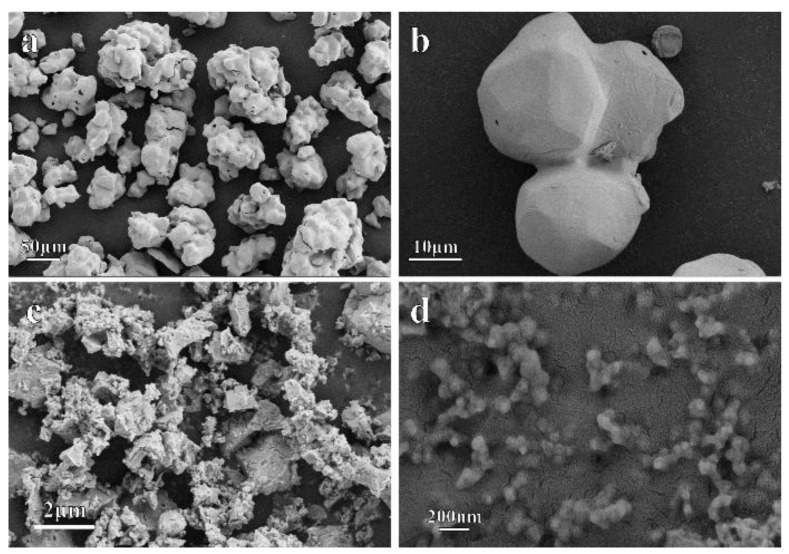
The typical morphological images of the Mn_3_O_4_ samples before (**a**,**b**) and after high-pressure torsion with 3 rotations (**c**,**d**).

The morphology and the microstructure of the Mn_3_O_4_ precursor and the severely-deformed samples were observed by using a Geminim SEM500 scanning electron microscope. The SEM photographs of the precursor and the HPT samples of Mn_3_O_4_ are shown in Figure 4. The precursor Mn_3_O_4_ micro-powder is irregular in shape with smooth surfaces and sizes ranging from several tens to several hundreds of microns, as shown in Figure 4a. Aggregation of the Mn_3_O_4_ powders is frequently observed, as shown in Figure 4a,b. A small piece of the Mn_3_O_4_ sample after HPT was ground and dispersed on the conductive sample holder for subsequent SEM observations. After HPT, most Mn_3_O_4_ particles are broken into small particles by the high applied pressures, as shown in Figure 4c. The size of the particles is usually no more than 1 µm, while the grain size should be much less than 1 µm for the presence of a large number of cracks/defects inside the particles. The rough surface and irregular shape of the samples after HPT indicates large fraction of grain boundaries in the sample. A detailed observation shows that the particle size of the sample after HPT is roughly less than 100 nm, as shown in Figure 4d.

**Figure 5:**
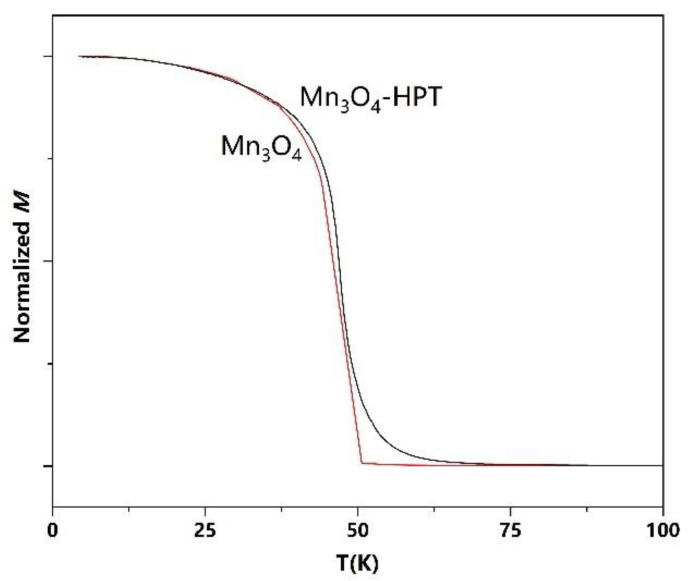
The M-T curves of the Mn_3_O_4_ precursor and Mn_3_O_4_ after HPT, respectively.

The magnetic properties of the samples were measured by using a Quantum Design physical property measurement system with increasing temperature. The temperature dependence of magnetization of the precursor and the severely deformed Mn_3_O_4_ samples (HPT for 3 turns) are shown in Figure 5. The magnetization of the precursor Mn_3_O_4_ decreases abruptly at ~42 K and vanishes at ~48 K, as shown in Figure 5. The sharp magnetization decrease at ~42 K is attributed to the Curie point of the ferrimagnetic Mn_3_O_4_. It should be noted that the temperatures of the sample may be lower than that of the environment during the heating process at 20 K/min because it needs time for thermal equilibrium, and this may result in a shift of the M-T curve to a higher temperature region. Estimated by using *dM/dT*, the magnetization of the Mn_3_O_4_ after HPT decreased abruptly at ~47 K, which is the *T_c_* of the sample. However, the magnetization of the HPT sample vanishes at ~65 K, which is higher than that of the precursor. We attribute the broadened ferrimagnetic-paramagnetic transition in the HPT sample to size effect. The severe deformation of Mn_3_O_4_ may produce a large number of defects and grain boundaries in the samples and thus a large fraction of uncompensated spins and local strains. It is known that the exchange coupling between Mn–Mn atoms is very sensitive to the atomic distance. The enhancement of the Curie temperature of Mn_3_O_4_ in nanoscale has been frequently observed in its low-dimensional systems [70,71,72]. The *T_c_* increases to 47 K in Mn_3_O_4_ nanorods and 45 K in Mn_3_O_4_/MnO nanoparticles, owing to finite scale and surface effects [71,72].

**Figure 6:**
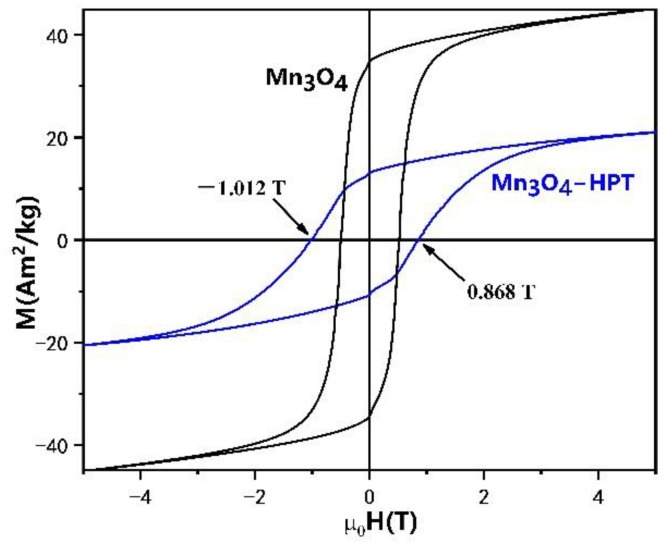
Magnetic hysteresis loops of the HPT Mn_3_O_4_ and Mn_3_O_4_ precursor measured at 5 K.

The magnetic hysteresis loops (M-H) of the Mn_3_O_4_ precursor and the severely deformed Mn_3_O_4_ were measured at 5 K, as shown in Figure 6. It should be noted that the M-H curves were measured after field cooling from room temperature. The coercivity of the undeformed Mn_3_O_4_ and the Mn_3_O_4_ after HPT for 3 turns at 5 K is 0.498 T and 1.007 T, respectively. The coercivity of the sample was doubled after HPT, owing to the significant grain size refinement of the Mn_3_O_4_ during severe deformation. The coercivity of the bulk HPT Mn_3_O_4_ is similar to that of the 1.05 T coercivity of the Mn_3_O_4_ nanoparticles prepared by laser ablation method [73].

The saturation magnetization *M_s_* of the Mn_3_O_4_ precursor and the severely deformed Mn_3_O_4_ is 45.1 Am^2^/kg and 21 Am^2^/kg, respectively. We attribute the decreased saturation magnetization in the HPT samples to the presence of large fraction of grain boundaries/defects and the increased fraction of the surface uncompensated spins in the samples. Detailed investigation shows that the M-H loops of the HPT sample shift to the negative field along the field axis, indicating the presence of an exchange bias in the sample. An exchange bias of 21 mT was observed in the precursors of Mn_3_O_4_. The exchange bias was elevated to 74 mT in Mn_3_O_4_-HPT samples. The exchange bias phenomenon usually occurs in the interface between magnetic phases with different magnetic orderings—ferromagnetic/antiferromagnetic interfaces, for instance. In this work, only ferrimagnetic Mn_3_O_4_ were detected by XRD measurement on our samples. However, we could not exclude the presence of trace amount of other manganese oxides, such as MnO. It is known that MnO is antiferromagnetic phase. The large fraction of defects and strains may also make the magnetic exchange coupling more complicated. The exchange bias can also be induced by the uncompensated spins.

## 7. Application of HPT to Magnetic Nitrides

We prepared nanocrystalline Mn_4_N bulk samples by using HPT from self-made Mn_4_N powders and studied the magnetic properties. In analogy to our previous work [65], the manganese powders were calcined at 823 K in flowing nitrogen (∼99.9wt%, 0.1 MPa) for 64 h to produce Mn_4_N powders. The Mn_4_N disc (φ = 1 cm) was severely deformed by HPT under a load of 300 kN for 3 turns of rotation at room temperature.

The XRD patterns of the as-prepared Mn_4_N powders could be indexed with Mn_4_N (JCPDS Card No. 89-3704) as the major phase and trace amount of MnO and Mn_2_N_0.86_, as shown in Figure 3c. The diffraction peaks of Mn_4_N are narrow and sharp, indicating large crystalline size of Mn_4_N. The presence of the trace amount of MnO was attributed to the limited vacuum and the limited purity of the nitrogen gas during the high-temperature reaction process. The spontaneous oxidation of Mn powders when exposed to air for a long time may also introduce a small amount of MnO into the samples. The formation of Mn_2_N_0.86_ was attributed to the in-equilibrium reaction between Mn and N_2_. It is known that a number of manganese nitrides, including *α*-, *β*-, *δ*-, *γ*-, *ε*-, *ζ*-, η-, and θ-phase, with varied nitrogen contents, could be formed [74]. Although no diffraction peaks from other phases of manganese nitrides or unreacted Mn could be found in the XRD patterns, we could not exclude their presence for limited sensitivity of the XRD techniques. The XRD patterns of the severely-deformed Mn_4_N after 3 turns of high-pressure torsion could be indexed with Mn_4_N and α-Mn, as shown in Figure 3d. It is interesting that the α-Mn phase was not detected in the precursor by using XRD, as seen in Figure 3c. We tend to believe that the unreacted Mn was wrapped by a thick layer of Mn_4_N during the nitriding process, and thus the Mn phase was not detected by XRD in the precursor. However, the Mn in the core region of the Mn_4_N particles may be exposed to the surface of the samples after severe deformation, and thus Mn was detected by XRD, as shown in Figure 3d. The diffraction peaks of Mn_4_N are significantly broadened due to the grain size refinement during the deformation process. It is hard to index Mn_2_N_0.86_ and MnO in Figure 3d because of the limited fraction of these phases and ultra-fine grain size of these phases after HPT. According to the Scherrer equation, the grain size of the Mn_4_N HPT samples is estimated to be 15~30 nm.

**Figure 7:**
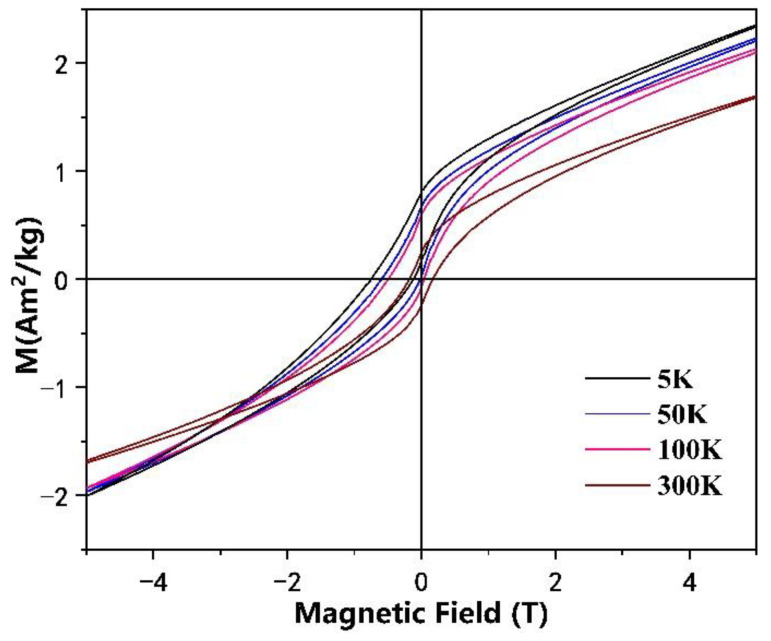
Magnetic hysteresis loops of the severely deformed Mn_4_N.

The M-H curves of the Mn_4_N after HPT were measured at 5 K, 50 K, 100 K, and 300 K, respectively, as shown in Figure 7. The coercivity of HPT-Mn_4_N reaches 0.75 T at 5 K. We attribute the increase in coercivity at 5 K to the small grain size. The magnetic hysteresis loop shifts to the negative field direction, indicating the presence of exchange bias in the samples. We ascribe the exchange bias to the interfacial exchange coupling between Mn_4_N and the antiferromagnetic phase (including Mn impurities). The exchange bias field reached up to 0.285 T in HPT-Mn_4_N at 5 K.

It is difficult to prepare high-density Sm-Fe-N for its low-decomposition temperature, and thus sintering method is not applicable for making bulk Sm-Fe-N. However, Sm–Fe–N bulk-disk-shaped Sm_2_Fe_17_N_x_ magnets were obtained by the HPT method for its low running temperature. After 3 turns of torsion, the sample has a maximum coercivity of 621.4 kA/m (7.7 kOe) and a remanent magnetization σr = 57.7 A·m^2^/kg. The high-pressure torsion induces the decomposition of Sm_2_Fe_17_N_x_ with the formation of α-Fe. [75].

## 8. Application of HPT to Magnetic Composites

HPT is demonstrated to be a promising processing method for exchange-spring magnetic materials in bulk form. The nanostructured Fe-SmCo exchange spring magnets were prepared by HPT-deformation on the powder mixtures of Fe and SmCo_5,_ over a broad chemical composition range. Magnetic measurements show hysteresis curves of an exchange-coupled nanocomposite at room temperature. The decoupling of Fe and SmCo_5_ was observed at low temperatures [58].

Ferromagnetic/antiferromagnetic Co/NiO composites at the 1:1 weight ratio were compacted by HPT, which reduces the crystallite size significantly and introduces large amounts of stacking faults in the metal [76]. Moreover, the fcc–Co transforms into hcp-Co after HPT. This mechanically driven phase transformation is usually accompanied by a strong reduction in the crystallite size and an increase of the micro-strains and stacking faults, resulting in an enhancement of the coercivity [76]. The magnetic coupling between ferromagnetic Co and antiferromagnetic NiO was confirmed by the presence of hysteresis loop shifts. Furthermore, the Vickers microhardness of Co/NiO powders compacted by HPT is almost twice that of pure Co subjected to HPT [76].

## 9. Challenges and Future Prospects for Applications of HPT in Magnetic Materials

As mentioned above, high-pressure torsion method is highly effective in producing ultrafine-grained magnetic materials and thus effective in tuning the magnetic parameters that are dependent on grain size. After HPT, most magnetic materials show reduced saturation magnetization and significantly enhanced coercivity, even though some exceptions could also be found due to occurrence of different mechanisms such as HPT-induced phase transformation. Moreover, the HPT method could also produce high-density nanocrystalline discs from bulk materials and/or powders, and this sometimes is useful for preparation of bulk magnetic materials with nano-size grains. Since the temperature for HPT processing is relatively low, this method might be used for fabrication of meta-stable phases and immiscible magnetic alloys and/or intermetallic materials that are not stable at elevated temperatures.

However, there are still several challenges that may limit the applications of HPT processing to magnetic materials. First of all, the size of the HPT products is limited, usually no more than several millimeters in thickness and approximately 1 cm or so in diameter. The size of the HPT samples is large enough for fundamental research but is difficult for industrial application, where usually needs large size products. Design of larger and more powerful HPT devices is helpful to produce larger samples but not enough [77]. Secondly, the rotation of the anvils in the HPT process makes it difficult to prepare magnetically well-aligned textures. Usually, the crystallographic orientations of the nano-grains in a bulk magnetic material have a substantial effect on the magnetic performance of the bulk samples, especially hard magnetic materials. We think that the orientation of the nano-grains may be improved to some extent by controlling the rotation angles of the anvil. Thirdly, the HPT process may result in the partial decomposition of some magnetic phases, and this is usually detrimental to the magnetic properties of the materials. The decomposition of the samples under HPT maybe improved by reducing the pressure applied or the rotation numbers. In summary, HPT is a powerful tool for the processing of magnetic materials and the tuning of magnetic properties.

## Figures and Tables

**Table 1:** The coercivity of the selected undeformed samples and HPT-deformed samples at room temperature (RT) or elevated temperatures.

Sample	Coercivity (A/m)	Sample	Coercivity (A/m)
Fe-3wt%Si/undeformed [20]	47	FeNi_3_ [7]	~990
Fe-3wt%Si-HPT-RT [20]	700	FeCo [7]	~6000
Fe-3wt%Si-HPT-723K [20]	842		
Fe-6.5wt%Si/undeformed [20]	60	Fe-0.45wt%C/undeformed [21]	~1100
Fe-6.5wt%Si-HPT-RT [20]	465	Fe-0.45wt%C-HPT [21]	~1800
Fe-6.5wt%Si-HPT-723K [20]	340		
Fe-17wt%Co-undeformed [20]	349	Cu-7%wtFe-HPT [22]	~8400
Fe-17wt%Co-HPT-RT [20]	1580	Cu-22wt%Fe-undeformed [23]	~15,900
Fe-17wt%Co-HPT-723K [20]	2492	Cu-22wt%Fe-HPT [23]	~24,600
Cu-10wt%Co-undeformed [23]	~12,000	MnAl-undeformed [24]	94,700
Cu-10wt%Co-HPT [23]	~30,000	MnAl-HPT [24]	470,000
Cu-2.2wt%Co-undeformed [25]	302	Fe-original [20]	150-RT
Cu-2.2wt%Co-HPT [25]	597	Fe-HPT [20]	1870-RT
Cu-4.9wt%Co-undeformed [25]	4220	Fe-HPT [20]	1035–723 K
Cu-4.9wt%Co-HPT [25]	17,900	Ni-original [20]	1806-RT
Co-5.6wt%Cu-undeformed [26]	1030	Ni-HPT [20]	1856-RT
Co-5.6wt% Cu-HPT [26]	9550	Ni-HPT [27]	5120
Co-13.6wt%Cu-undeformed [26]	135	Co [28]	16,716
Co-13.6 wt% Cu-HPT [26]	8280	Co-HPT [28]	27,860

**Table 2:** The coercivity of the selected ternary magnetic alloys before and/or after HPT.

Sample	Coercivity (A/m)	Sample	Coercivity (A/m)
Nd_9_Fe_85_B_6_ melt-spun [52]	405,960	47wt%Fe-53wt%SmCo_5_-HPT [58]	~159,200–300 K
Nd_9_Fe_85_B_6_-HPT [52]	429,840		~278,600–8 K
α-Fe/Nd_2_Fe_14_B [50]	~238,800–923 K	α-Fe/Nd_2_Fe_14_B [51]	366,140
α-Fe/Nd_2_Fe_14_B-HPT [50]	~318,400–923 K	α-Fe/Nd_2_Fe_14_B-HPT [51]	509,440
Co_80_Zr_16_B_4_-HPT [59]	89,550		

## Data Availability

Data sharing is not applicable for this article.
